# Writing a Scientific Review Article: Comprehensive Insights for Beginners

**DOI:** 10.1155/2024/7822269

**Published:** 2024-01-17

**Authors:** Ayodeji Amobonye, Japareng Lalung, Gift Mheta, Santhosh Pillai

**Affiliations:** ^1^Department of Biotechnology and Food Science, Faculty of Applied Sciences, Durban University of Technology, P.O. Box 1334, KwaZulu-Natal, Durban 4000, South Africa; ^2^Writing Centre, Durban University of Technology, P.O. Box 1334 KwaZulu-Natal, Durban 4000, South Africa; ^3^School of Industrial Technology, Universiti Sains Malaysia, Gelugor 11800, Pulau Pinang, Malaysia

## Abstract

Review articles present comprehensive overview of relevant literature on specific themes and synthesise the studies related to these themes, with the aim of strengthening the foundation of knowledge and facilitating theory development. The significance of review articles in science is immeasurable as both students and researchers rely on these articles as the starting point for their research. Interestingly, many postgraduate students are expected to write review articles for journal publications as a way of demonstrating their ability to contribute to new knowledge in their respective fields. However, there is no comprehensive instructional framework to guide them on how to analyse and synthesise the literature in their niches into publishable review articles. The dearth of ample guidance or explicit training results in students having to learn all by themselves, usually by trial and error, which often leads to high rejection rates from publishing houses. Therefore, this article seeks to identify these challenges from a beginner's perspective and strives to plug the identified gaps and discrepancies. Thus, the purpose of this paper is to serve as a systematic guide for emerging scientists and to summarise the most important information on how to write and structure a publishable review article.

## 1. Introduction

Early scientists, spanning from the Ancient Egyptian civilization to the Scientific Revolution of the 16^th^/17^th^ century, based their research on intuitions, personal observations, and personal insights. Thus, less time was spent on background reading as there was not much literature to refer to. This is well illustrated in the case of Sir Isaac Newton's apple tree and the theory of gravity, as well as Gregor Mendel's pea plants and the theory of inheritance. However, with the astronomical expansion in scientific knowledge and the emergence of the information age in the last century, new ideas are now being built on previously published works, thus the periodic need to appraise the huge amount of already published literature [1]. According to Birkle et al. [2], the Web of Science—an authoritative database of research publications and citations—covered more than 80 million scholarly materials. Hence, a critical review of prior and relevant literature is indispensable for any research endeavour as it provides the necessary framework needed for synthesising new knowledge and for highlighting new insights and perspectives [3].

Review papers are generally considered secondary research publications that sum up already existing works on a particular research topic or question and relate them to the current status of the topic. This makes review articles distinctly different from scientific research papers. While the primary aim of the latter is to develop new arguments by reporting original research, the former is focused on summarising and synthesising previous ideas, studies, and arguments, without adding new experimental contributions. Review articles basically describe the content and quality of knowledge that are currently available, with a special focus on the significance of the previous works. To this end, a review article cannot simply reiterate a subject matter, but it must contribute to the field of knowledge by synthesising available materials and offering a scholarly critique of theory [4]. Typically, these articles critically analyse both quantitative and qualitative studies by scrutinising experimental results, the discussion of the experimental data, and in some instances, previous review articles to propose new working theories. Thus, a review article is more than a mere exhaustive compilation of all that has been published on a topic; it must be a balanced, informative, perspective, and unbiased compendium of previous studies which may also include contrasting findings, inconsistencies, and conventional and current views on the subject [5].

Hence, the essence of a review article is measured by what is achieved, what is discovered, and how information is communicated to the reader [6]. According to Steward [7], a good literature review should be analytical, critical, comprehensive, selective, relevant, synthetic, and fully referenced. On the other hand, a review article is considered to be inadequate if it is lacking in focus or outcome, overgeneralised, opinionated, unbalanced, and uncritical [7]. Most review papers fail to meet these standards and thus can be viewed as mere summaries of previous works in a particular field of study. In one of the few studies that assessed the quality of review articles, none of the 50 papers that were analysed met the predefined criteria for a good review [8]. However, beginners must also realise that there is no bad writing in the true sense; there is only writing in evolution and under refinement. Literally, every piece of writing can be improved upon, right from the first draft until the final published manuscript. Hence, a paper can only be referred to as bad and unfixable when the author is not open to corrections or when the writer gives up on it.

According to Peat et al. [9], “everything is easy when you know how,” a maxim which applies to scientific writing in general and review writing in particular. In this regard, the authors emphasized that the writer should be open to learning and should also follow established rules instead of following a blind trial-and-error approach. In contrast to the popular belief that review articles should only be written by experienced scientists and researchers, recent trends have shown that many early-career scientists, especially postgraduate students, are currently expected to write review articles during the course of their studies. However, these scholars have little or no access to formal training on how to analyse and synthesise the research literature in their respective fields [10]. Consequently, students seeking guidance on how to write or improve their literature reviews are less likely to find published works on the subject, particularly in the science fields. Although various publications have dealt with the challenges of searching for literature, or writing literature reviews for dissertation/thesis purposes, there is little or no information on how to write a comprehensive review article for publication. In addition to the paucity of published information to guide the potential author, the lack of understanding of what constitutes a review paper compounds their challenges. Thus, the purpose of this paper is to serve as a guide for writing review papers for journal publishing. This work draws on the experience of the authors to assist early-career scientists/researchers in the “hard skill” of authoring review articles. Even though there is no single path to writing scientifically, or to writing reviews in particular, this paper attempts to simplify the process by looking at this subject from a beginner's perspective. Hence, this paper highlights the differences between the types of review articles in the sciences while also explaining the needs and purpose of writing review articles. Furthermore, it presents details on how to search for the literature as well as how to structure the manuscript to produce logical and coherent outputs. It is hoped that this work will ease prospective scientific writers into the challenging but rewarding art of writing review articles.

## 2. Benefits of Review Articles to the Author

Analysing literature gives an overview of the “WHs”: WHat has been reported in a particular field or topic, WHo the key writers are, WHat are the prevailing theories and hypotheses, WHat questions are being asked (and answered), and WHat methods and methodologies are appropriate and useful [11]. For new or aspiring researchers in a particular field, it can be quite challenging to get a comprehensive overview of their respective fields, especially the historical trends and what has been studied previously. As such, the importance of review articles to knowledge appraisal and contribution cannot be overemphasised, which is reflected in the constant demand for such articles in the research community. However, it is also important for the author, especially the first-time author, to recognise the importance of his/her investing time and effort into writing a quality review article.

Generally, literature reviews are undertaken for many reasons, mainly for publication and for dissertation purposes. The major purpose of literature reviews is to provide direction and information for the improvement of scientific knowledge. They also form a significant component in the research process and in academic assessment [12]. There may be, however, a thin line between a dissertation literature review and a published review article, given that with some modifications, a literature review can be transformed into a legitimate and publishable scholarly document. According to Gülpınar and Güçlü [6], the basic motivation for writing a review article is to make a comprehensive synthesis of the most appropriate literature on a specific research inquiry or topic. Thus, conducting a literature review assists in demonstrating the author's knowledge about a particular field of study, which may include but not be limited to its history, theories, key variables, vocabulary, phenomena, and methodologies [10]. Furthermore, publishing reviews is beneficial as it permits the researchers to examine different questions and, as a result, enhances the depth and diversity of their scientific reasoning [1]. In addition, writing review articles allows researchers to share insights with the scientific community while identifying knowledge gaps to be addressed in future research. The review writing process can also be a useful tool in training early-career scientists in leadership, coordination, project management, and other important soft skills necessary for success in the research world [13]. Another important reason for authoring reviews is that such publications have been observed to be remarkably influential, extending the reach of an author in multiple folds of what can be achieved by primary research papers [1]. The trend in science is for authors to receive more citations from their review articles than from their original research articles. According to Miranda and Garcia-Carpintero [14], review articles are, on average, three times more frequently cited than original research articles; they also asserted that a 20% increase in review authorship could result in a 40–80% increase in citations of the author. As a result, writing reviews can significantly impact a researcher's citation output and serve as a valuable channel to reach a wider scientific audience. In addition, the references cited in a review article also provide the reader with an opportunity to dig deeper into the topic of interest. Thus, review articles can serve as a valuable repository for consultation, increasing the visibility of the authors and resulting in more citations.

## 3. Types of Review Articles

The first step in writing a good literature review is to decide on the particular type of review to be written; hence, it is important to distinguish and understand the various types of review articles. Although scientific review articles have been classified according to various schemes, however, they are broadly categorised into narrative reviews, systematic reviews, and meta-analyses [15]. It was observed that more authors—as well as publishers—were leaning towards systematic reviews and meta-analysis while downplaying narrative reviews; however, the three serve different aims and should all be considered equally important in science [1]. Bibliometric reviews and patent reviews, which are closely related to meta-analysis, have also gained significant attention recently. However, from another angle, a review could also be of two types. In the first class, authors could deal with a widely studied topic where there is already an accumulated body of knowledge that requires analysis and synthesis [3]. At the other end of the spectrum, the authors may have to address an emerging issue that would benefit from exposure to potential theoretical foundations; hence, their contribution would arise from the fresh theoretical foundations proposed in developing a conceptual model [3].

### 3.1. Narrative Reviews

Narrative reviewers are mainly focused on providing clarification and critical analysis on a particular topic or body of literature through interpretative synthesis, creativity, and expert judgement. According to Green et al. [16], a narrative review can be in the form of editorials, commentaries, and narrative overviews. However, editorials and commentaries are usually expert opinions; hence, a beginner is more likely to write a narrative overview, which is more general and is also referred to as an unsystematic narrative review. Similarly, the literature review section of most dissertations and empirical papers is typically narrative in nature. Typically, narrative reviews combine results from studies that may have different methodologies to address different questions or to formulate a broad theoretical formulation [1]. They are largely integrative as strong focus is placed on the assimilation and synthesis of various aspects in the review, which may involve comparing and contrasting research findings or deriving structured implications [17]. In addition, they are also qualitative studies because they do not follow strict selection processes; hence, choosing publications is relatively more subjective and unsystematic [18]. However, despite their popularity, there are concerns about their inherent subjectivity. In many instances, when the supporting data for narrative reviews are examined more closely, the evaluations provided by the author(s) become quite questionable [19]. Nevertheless, if the goal of the author is to formulate a new theory that connects diverse strands of research, a narrative method is most appropriate.

### 3.2. Systematic Reviews

In contrast to narrative reviews, which are generally descriptive, systematic reviews employ a systematic approach to summarise evidence on research questions. Hence, systematic reviews make use of precise and rigorous criteria to identify, evaluate, and subsequently synthesise all relevant literature on a particular topic [12, 20]. As a result, systematic reviews are more likely to inspire research ideas by identifying knowledge gaps or inconsistencies, thus helping the researcher to clearly define the research hypotheses or questions [21]. Furthermore, systematic reviews may serve as independent research projects in their own right, as they follow a defined methodology to search and combine reliable results to synthesise a new database that can be used for a variety of purposes [22]. Typically, the peculiarities of the individual reviewer, different search engines, and information databases used all ensure that no two searches will yield the same systematic results even if the searches are conducted simultaneously and under identical criteria [11]. Hence, attempts are made at standardising the exercise via specific methods that would limit bias and chance effects, prevent duplications, and provide more accurate results upon which conclusions and decisions can be made.

The most established of these methods is the PRISMA (Preferred Reporting Items for Systematic Reviews and Meta-Analyses) guidelines which objectively defined statements, guidelines, reporting checklists, and flowcharts for undertaking systematic reviews as well as meta-analysis [23]. Though mainly designed for research in medical sciences, the PRISMA approach has gained wide acceptance in other fields of science and is based on eight fundamental propositions. These include the explicit definition of the review question, an unambiguous outline of the study protocol, an objective and exhaustive systematic review of reputable literature, and an unambiguous identification of included literature based on defined selection criteria [24]. Other considerations include an unbiased appraisal of the quality of the selected studies (literature), organic synthesis of the evidence of the study, preparation of the manuscript based on the reporting guidelines, and periodic update of the review as new data emerge [24]. Other methods such as PRISMA-P (Preferred Reporting Items for Systematic review and Meta-Analysis Protocols), MOOSE (Meta-analysis Of Observational Studies in Epidemiology), and ROSES (Reporting Standards for Systematic Evidence Syntheses) have since been developed for systematic reviews (and meta-analysis), with most of them being derived from PRISMA.

Consequently, systematic reviews—unlike narrative reviews—must contain a methodology section which in addition to all that was highlighted above must fully describe the precise criteria used in formulating the research question and setting the inclusion or exclusion criteria used in selecting/accessing the literature. Similarly, the criteria for evaluating the quality of the literature included in the review as well as for analysing, synthesising, and disseminating the findings must be fully described in the methodology section.

### 3.3. Meta-Analysis

Meta-analyses are considered as more specialised forms of systematic reviews. Generally, they combine the results of many studies that use similar or closely related methods to address the same question or share a common quantitative evaluation method [25]. However, meta-analyses are also a step higher than other systematic reviews as they are focused on numerical data and involve the use of statistics in evaluating different studies and synthesising new knowledge. The major advantage of this type of review is the increased statistical power leading to more reliable results for inferring modest associations and a more comprehensive understanding of the true impact of a research study [26]. Unlike in traditional systematic reviews, research topics covered in meta-analyses must be mature enough to allow the inclusion of sufficient homogeneous empirical research in terms of subjects, interventions, and outcomes [27, 28].

Being an advanced form of systematic review, meta-analyses must also have a distinct methodology section; hence, the standard procedures involved in the traditional systematic review (especially PRISMA) also apply in meta-analyses [23]. In addition to the common steps in formulating systematic reviews, meta-analyses are required to describe how nested and missing data are handled, the effect observed in each study, the confidence interval associated with each synthesised effect, and any potential for bias presented within the sample(s) [17]. According to Paul and Barari [28], a meta-analysis must also detail the final sample, the meta-analytic model, and the overall analysis, moderator analysis, and software employed. While the overall analysis involves the statistical characterization of the relationships between variables in the meta-analytic framework and their significance, the moderator analysis defines the different variables that may affect variations in the original studies [28, 29]. It must also be noted that the accuracy and reliability of meta-analyses have both been significantly enhanced by the incorporation of statistical approaches such as Bayesian analysis [30], network analysis [31], and more recently, machine learning [32].

### 3.4. Bibliometric Review

A bibliometric review, commonly referred to as bibliometric analysis, is a systematic evaluation of published works within a specific field or discipline [33]. This bibliometric methodology involves the use of quantitative methods to analyse bibliometric data such as the characteristics and numbers of publications, units of citations, authorship, co-authorship, and journal impact factors [34]. Academics use bibliometric analysis with different objectives in mind, which includes uncovering emerging trends in article and journal performance, elaborating collaboration patterns and research constituents, evaluating the impact and influence of particular authors, publications, or research groups, and highlighting the intellectual framework of a certain field [35]. It is also used to inform policy and decision-making. Similarly to meta-analysis, bibliometric reviews rely upon quantitative techniques, thus avoiding the interpretation bias that could arise from the qualitative techniques of other types of reviews [36]. However, while bibliometric analysis synthesises the bibliometric and intellectual structure of a field by examining the social and structural linkages between various research parts, meta-analysis focuses on summarising empirical evidence by probing the direction and strength of effects and relationships among variables, especially in open research questions [37, 38]. However, similarly to systematic review and meta-analysis, a bibliometric review also requires a well-detailed methodology section. The amount of data to be analysed in bibliometric analysis is quite massive, running to hundreds and tens of thousands in some cases. Although the data are objective in nature (e.g., number of citations and publications and occurrences of keywords and topics), the interpretation is usually carried out through both objective (e.g., performance analysis) and subjective (e.g., thematic analysis) evaluations [35]. However, the invention and availability of bibliometric software such as BibExcel, Gephi, Leximancer, and VOSviewer and scientific databases such as Dimensions, Web of Science, and Scopus have made this type of analysis more feasible.

### 3.5. Patent Review

Patent reviews provide a comprehensive analysis and critique of a specific patent or a group of related patents, thus presenting a concise understanding of the technology or innovation that is covered by the patent [39]. This type of article is useful for researchers as it also enhances their understanding of the legal, technical, and commercial aspects of an intellectual property/innovation; in addition, it is also important for stakeholders outside the research community including IP (intellectual property) specialists, legal professionals, and technology-transfer officers [40]. Typically, patent reviews encompass the scope, background, claims, legal implications, technical specifications, and potential commercial applications of the patent(s). The article may also include a discussion of the patent's strengths and weaknesses, as well as its potential impact on the industry or field in which it operates. Most times, reviews are time specified, they may be regionalised, and the data are usually retrieved via patent searches on databases such as that of the European Patent Office (https://www.epo.org/searching.html), United States Patent and Trademark Office (https://patft.uspto.gov/), the World Intellectual Property Organization's PATENTSCOPE (https://patentscope.wipo.int/search/en/structuredSearch.jsf), Google Patent (https://www.google.com/?tbm=pts), and China National Intellectual Property Administration (https://pss-system.cponline.cnipa.gov.cn/conventionalSearch). According to Cerimi et al. [41], the retrieved data and analysed may include the patent number, patent status, filing date, application date, grant dates, inventor, assignee, and pending applications. While data analysis is usually carried out by general data software such as Microsoft Excel, an intelligence software solely dedicated to patent research and analysis, Orbit Intelligence has been found to be more efficient [39]. It is also mandatory to include a methodology section in a patent review, and this should be explicit, thorough, and precise to allow a clear understanding of how the analysis was carried out and how the conclusions were arrived at.

## 4. Searching Literature

One of the most challenging tasks in writing a review article on a subject is the search for relevant literature to populate the manuscript as the author is required to garner information from an endless number of sources. This is even more challenging as research outputs have been increasing astronomically, especially in the last decade, with thousands of new articles published annually in various fields. It is therefore imperative that the author must not only be aware of the overall trajectory in a field of investigation but must also be cognizant of recent studies so as not to publish outdated research or review articles. Basically, the search for the literature involves a coherent conceptual structuring of the topic itself and a thorough collation of evidence under the common themes which might reflect the histories, conflicts, standoffs, revolutions, and/or evolutions in the field [7]. To start the search process, the author must carefully identify and select broad keywords relevant to the subject; subsequently, the keywords should be developed to refine the search into specific subheadings that would facilitate the structure of the review.

Two main tactics have been identified for searching the literature, namely, systematic and snowballing [42]. The systematic approach involves searching literature with specific keywords (for example, cancer, antioxidant, and nanoparticles), which leads to an almost unmanageable and overwhelming list of possible sources [43]. The snowballing approach, however, involves the identification of a particular publication, followed by the compilation of a bibliography of articles based on the reference list of the identified publication [44]. Many times, it might be necessary to combine both approaches, but irrespective, the author must keep an accurate track and record of papers cited in the search. A simple and efficient strategy for populating the bibliography of review articles is to go through the abstract (and sometimes the conclusion) of a paper; if the abstract is related to the topic of discourse, the author might go ahead and read the entire article; otherwise, he/she is advised to move on [45]. Winchester and Salji [5] noted that to learn the background of the subject/topic to be reviewed, starting literature searches with academic textbooks or published review articles is imperative, especially for beginners. Furthermore, it would also assist in compiling the list of keywords, identifying areas of further exploration, and providing a glimpse of the current state of the research. However, past reviews ideally are not to serve as the foundation of a new review as they are written from someone else's viewpoint, which might have been tainted with some bias. Fortunately, the accessibility and search for the literature have been made relatively easier than they were a few decades ago as the current information age has placed an enormous volume of knowledge right at our fingertips [46]. Nevertheless, when gathering the literature from the Internet, authors should exercise utmost caution as much of the information may not be verified or peer-reviewed and thus may be unregulated and unreliable. For instance, Wikipedia, despite being a large repository of information with more than 6.7 million articles in the English language alone, is considered unreliable for scientific literature reviews, due to its openness to public editing [47]. However, in addition to peer-reviewed journal publications—which are most ideal—reviews can also be drawn from a wide range of other sources such as technical documents, in-house reports, conference abstracts, and conference proceedings. Similarly, “Google Scholar”—as against “Google” and other general search engines—is more appropriate as its searches are restricted to only academic articles produced by scholarly societies or/and publishers [48]. Furthermore, the various electronic databases, such as ScienceDirect, Web of Science, PubMed, and MEDLINE, many of which focus on specific fields of research, are also ideal options [49]. Advancement in computer indexing has remarkably expanded the ease and ability to search large databases for every potentially relevant article. In addition to searching by topic, literature search can be modified by time; however, there must be a balance between old papers and recent ones. The general consensus in science is that publications less than five years old are considered recent.

It is important, especially in systematic reviews and meta-analyses, that the specific method of running the computer searches be properly documented as there is the need to include this in the method (methodology) section of such papers. Typically, the method details the keywords, databases explored, search terms used, and the inclusion/exclusion criteria applied in the selection of data and any other specific decision/criteria. All of these will ensure the reproducibility and thoroughness of the search and the selection procedure. However, Randolph [10] noted that Internet searches might not give the exhaustive list of articles needed for a review article; hence, it is advised that authors search through the reference lists of articles that were obtained initially from the Internet search. After determining the relevant articles from the list, the author should read through the references of these articles and repeat the cycle until saturation is reached [10]. After populating the articles needed for the literature review, the next step is to analyse them individually and in their whole entirety. A systematic approach to this is to identify the key information within the papers, examine them in depth, and synthesise original perspectives by integrating the information and making inferences based on the findings. In this regard, it is imperative to link one source to the other in a logical manner, for instance, taking note of studies with similar methodologies, papers that agree, or results that are contradictory [42].

## 5. Structuring the Review Article

### 5.1. Title

The title and abstract are the main selling points of a review article, as most readers will only peruse these two elements and usually go on to read the full paper if they are drawn in by either or both of the two. Tullu [50] recommends that the title of a scientific paper “should be descriptive, direct, accurate, appropriate, interesting, concise, precise, unique, and not be misleading.” In addition to providing “just enough details” to entice the reader, words in the titles are also used by electronic databases, journal websites, and search engines to index and retrieve a particular paper during a search [51]. Titles are of different types and must be chosen according to the topic under review. They are generally classified as descriptive, declarative, or interrogative and can also be grouped into compound, nominal, or full-sentence titles [50]. The subject of these categorisations has been extensively discussed in many articles; however, the reader must also be aware of the compound titles, which usually contain a main title and a subtitle. Typically, subtitles provide additional context—to the main title—and they may specify the geographic scope of the research, research methodology, or sample size [52].

Just like primary research articles, there are many debates about the optimum length of a review article's title. However, the general consensus is to keep the title as brief as possible while not being too general. A title length between 10 and 15 words is recommended, since longer titles can be more challenging to comprehend. Paiva et al. [53] observed that articles which contain 95 characters or less get more views and citations. However, emphasis must be placed on conciseness as the audience will be more satisfied if they can understand what exactly the review has contributed to the field, rather than just a hint about the general topic area. Authors should also endeavour to stick to the journal's specific requirements, especially regarding the length of the title and what they should or should not contain [9]. Thus, avoidance of filler words such as “a review on/of,” “an observation of,” or “a study of” is a very simple way to limit title length. In addition, abbreviations or acronyms should be avoided in the title, except the standard or commonly interpreted ones such as AIDS, DNA, HIV, and RNA. In summary, to write an effective title, the authors should consider the following points. What is the paper about? What was the methodology used? What were the highlights and major conclusions? Subsequently, the author should list all the keywords from these answers, construct a sentence from these keywords, and finally delete all redundant words from the sentence title. It is also possible to gain some ideas by scanning indices and article titles in major journals in the field. It is important to emphasise that a title is not chosen and set in stone, and the title is most likely to be continually revised and adjusted until the end of the writing process.

### 5.2. Abstract

The abstract, also referred to as the synopsis, is a summary of the full research paper; it is typically independent and can stand alone. For most readers, a publication does not exist beyond the abstract, partly because abstracts are often the only section of a paper that is made available to the readers at no cost, whereas the full paper may attract a payment or subscription [54]. Thus, the abstract is supposed to set the tone for the few readers who wish to read the rest of the paper. It has also been noted that the abstract gives the first impression of a research work to journal editors, conference scientific committees, or referees, who might outright reject the paper if the abstract is poorly written or inadequate [50]. Hence, it is imperative that the abstract succinctly represents the entire paper and projects it positively. Just like the title, abstracts have to be balanced, comprehensive, concise, functional, independent, precise, scholarly, and unbiased and not be misleading [55]. Basically, the abstract should be formulated using keywords from all the sections of the main manuscript. Thus, it is pertinent that the abstract conveys the focus, key message, rationale, and novelty of the paper without any compromise or exaggeration. Furthermore, the abstract must be consistent with the rest of the paper; as basic as this instruction might sound, it is not to be taken for granted. For example, a study by Vrijhoef and Steuten [56] revealed that 18–68% of 264 abstracts from some scientific journals contained information that was inconsistent with the main body of the publications.

Abstracts can either be structured or unstructured; in addition, they can further be classified as either descriptive or informative. Unstructured abstracts, which are used by many scientific journals, are free flowing with no predefined subheadings, while structured abstracts have specific subheadings/subsections under which the abstract needs to be composed. Structured abstracts have been noted to be more informative and are usually divided into subsections which include the study background/introduction, objectives, methodology design, results, and conclusions [57]. No matter the style chosen, the author must carefully conform to the instructions provided by the potential journal of submission, which may include but are not limited to the format, font size/style, word limit, and subheadings [58]. The word limit for abstracts in most scientific journals is typically between 150 and 300 words. It is also a general rule that abstracts do not contain any references whatsoever.

Typically, an abstract should be written in the active voice, and there is no such thing as a perfect abstract as it could always be improved on. It is advised that the author first makes an initial draft which would contain all the essential parts of the paper, which could then be polished subsequently. The draft should begin with a brief background which would lead to the research questions. It might also include a general overview of the methodology used (if applicable) and importantly, the major results/observations/highlights of the review paper. The abstract should end with one or few sentences about any implications, perspectives, or future research that may be developed from the review exercise. Finally, the authors should eliminate redundant words and edit the abstract to the correct word count permitted by the journal [59]. It is always beneficial to read previous abstracts published in the intended journal, related topics/subjects from other journals, and other reputable sources. Furthermore, the author should endeavour to get feedback on the abstract especially from peers and co-authors. As the abstract is the face of the whole paper, it is best that it is the last section to be finalised, as by this time, the author would have developed a clearer understanding of the findings and conclusions of the entire paper.

### 5.3. Graphical Abstracts

Since the mid-2000s, an increasing number of journals now require authors to provide a graphical abstract (GA) in addition to the traditional written abstract, to increase the accessibility of scientific publications to readers [60]. A study showed that publications with GA performed better than those without it, when the abstract views, total citations, and downloads were compared [61]. However, the GA should provide “a single, concise pictorial, and visual summary of the main findings of an article” [62]. Although they are meant to be a stand-alone summary of the whole paper, it has been noted that they are not so easily comprehensible without having read through the traditionally written abstract [63]. It is important to note that, like traditional abstracts, many reputable journals require GAs to adhere to certain specifications such as colour, dimension, quality, file size, and file format (usually JPEG/JPG, PDF, PNG, or TIFF). In addition, it is imperative to use engaging and accurate figures, all of which must be synthesised in order to accurately reflect the key message of the paper. Currently, there are various online or downloadable graphical tools that can be used for creating GAs, such as Microsoft Paint or PowerPoint, Mindthegraph, ChemDraw, CorelDraw, and BioRender.

### 5.4. Keywords

As a standard practice, journals require authors to select 4–8 keywords (or phrases), which are typically listed below the abstract. A good set of keywords will enable indexers and search engines to find relevant papers more easily and can be considered as a very concise abstract [64]. According to Dewan and Gupta [51], the selection of appropriate keywords will significantly enhance the retrieval, accession, and consequently, the citation of the review paper. Ideally, keywords can be variants of the terms/phrases used in the title, the abstract, and the main text, but they should ideally not be the exact words in the main title. Choosing the most appropriate keywords for a review article involves listing down the key terms and phrases in the article, including abbreviations. Subsequently, a quick review of the glossary/vocabulary/term list or indexing standard in the specific discipline will assist in selecting the best and most precise keywords that match those used in the databases from the list drawn. In addition, the keywords should not be broad or general terms (e.g., DNA, biology, and enzymes) but must be specific to the field or subfield of study as well as to the particular paper [65].

### 5.5. Introduction

The introduction of an article is the first major section of the manuscript, and it presents basic information to the reader without compelling them to study past publications. In addition, the introduction directs the reader to the main arguments and points developed in the main body of the article while clarifying the current state of knowledge in that particular area of research [12]. The introduction part of a review article is usually sectionalised into background information, a description of the main topic and finally a statement of the main purpose of the review [66]. Authors may begin the introduction with brief general statements—which provide background knowledge on the subject matter—that lead to more specific ones [67]. It is at this point that the reader's attention must be caught as the background knowledge must highlight the importance and justification for the subject being discussed, while also identifying the major problem to be addressed [68]. In addition, the background should be broad enough to attract even nonspecialists in the field to maximise the impact and widen the reach of the article. All of these should be done in the light of current literature; however, old references may also be used for historical purposes. A very important aspect of the introduction is clearly stating and establishing the research problem(s) and how a review of the particular topic contributes to those problem(s). Thus, the research gap which the paper intends to fill, the limitations of previous works and past reviews, if available, and the new knowledge to be contributed must all be highlighted. Inadequate information and the inability to clarify the problem will keep readers (who have the desire to obtain new information) from reading beyond the introduction [69]. It is also pertinent that the author establishes the purpose of reviewing the literature and defines the scope as well as the major synthesised point of view. Furthermore, a brief insight into the criteria used to select, evaluate, and analyse the literature, as well as the outline or sequence of the review, should be provided in the introduction. Subsequently, the specific objectives of the review article must be presented. The last part of the “introduction” section should focus on the solution, the way forward, the recommendations, and the further areas of research as deduced from the whole review process. According to DeMaria [70], clearly expressed or recommended solutions to an explicitly revealed problem are very important for the wholesomeness of the “introduction” section. It is believed that following these steps will give readers the opportunity to track the problems and the corresponding solution from their own perspective in the light of current literature. As against some suggestions that the introduction should be written only in present tenses, it is also believed that it could be done with other tenses in addition to the present tense. In this regard, general facts should be written in the present tense, specific research/work should be in the past tense, while the concluding statement should be in the past perfect or simple past. Furthermore, many of the abbreviations to be used in the rest of the manuscript and their explanations should be defined in this section.

### 5.6. Methodology

Writing a review article is equivalent to conducting a research study, with the information gathered by the author (reviewer) representing the data. Like all major studies, it involves conceptualisation, planning, implementation, and dissemination [71], all of which may be detailed in a methodology section, if necessary. Hence, the methodological section of a review paper (which can also be referred to as the review protocol) details how the relevant literature was selected and how it was analysed as well as summarised. The selection details may include, but are not limited to, the database consulted and the specific search terms used together with the inclusion/exclusion criteria. As earlier highlighted in Section 3, a description of the methodology is required for all types of reviews except for narrative reviews. This is partly because unlike narrative reviews, all other review articles follow systematic approaches which must ensure significant reproducibility [72]. Therefore, where necessary, the methods of data extraction from the literature and data synthesis must also be highlighted as well. In some cases, it is important to show how data were combined by highlighting the statistical methods used, measures of effect, and tests performed, as well as demonstrating heterogeneity and publication bias [73].

The methodology should also detail the major databases consulted during the literature search, e.g., Dimensions, ScienceDirect, Web of Science, MEDLINE, and PubMed. For meta-analysis, it is imperative to highlight the software and/or package used, which could include Comprehensive Meta-Analysis, OpenMEE, Review Manager (RevMan), Stata, SAS, and R Studio. It is also necessary to state the mathematical methods used for the analysis; examples of these include the Bayesian analysis, the Mantel–Haenszel method, and the inverse variance method. The methodology should also state the number of authors that carried out the initial review stage of the study, as it has been recommended that at least two reviews should be done blindly and in parallel, especially when it comes to the acquisition and synthesis of data [74]. Finally, the quality and validity assessment of the publication used in the review must be stated and well clarified [73].

### 5.7. Main Body of the Review

Ideally, the main body of a publishable review should answer these questions: What is new (contribution)? Why so (logic)? So what (impact)? How well it is done (thoroughness)? The flow of the main body of a review article must be well organised to adequately maintain the attention of the readers as well as guide them through the section. It is recommended that the author should consider drawing a conceptual scheme of the main body first, using methods such as mind-mapping. This will help create a logical flow of thought and presentation, while also linking the various sections of the manuscript together. According to Moreira [75], “reports do not simply yield their findings, rather reviewers make them yield,” and thus, it is the author's responsibility to transform “resistant” texts into “docile” texts. Hence, after the search for the literature, the essential themes and key concepts of the review paper must be identified and synthesised together. This synthesis primarily involves creating hypotheses about the relationships between the concepts with the aim of increasing the understanding of the topic being reviewed. The important information from the various sources should not only be summarised, but the significance of studies must be related back to the initial question(s) posed by the review article. Furthermore, MacLure [76] stated that data are not just to be plainly “extracted intact” and “used exactly as extracted,” but must be modified, reconfigured, transformed, transposed, converted, tabulated, graphed, or manipulated to enable synthesis, combination, and comparison. Therefore, different pieces of information must be extracted from the reports in which they were previously deposited and then refined into the body of the new article [75]. To this end, adequate comparison and combination might require that “qualitative data be quantified” or/and “quantitative data may be qualitized” [77]. In order to accomplish all of these goals, the author may have to transform, paraphrase, generalize, specify, and reorder the text [78]. For comprehensiveness, the body paragraphs should be arranged in a similar order as it was initially stated in the abstract or/and introduction. Thus, the main body could be divided into thematic areas, each of which could be independently comprehensive and treated as a mini review. Similarly, the sections can also be arranged chronologically depending on the focus of the review. Furthermore, the abstractions should proceed from a wider general view of the literature being reviewed and then be narrowed down to the specifics. In the process, deep insights should also be provided between the topic of the review and the wider subject area, e.g., fungal enzymes and enzymes in general. The abstractions must also be discussed in more detail by presenting more specific information from the identified sources (with proper citations of course!). For example, it is important to identify and highlight contrary findings and rival interpretations as well as to point out areas of agreement or debate among different bodies of literature. Often, there are previous reviews on the same topic/concept; however, this does not prevent a new author from writing one on the same topic, especially if the previous reviews were written many years ago. However, it is important that the body of the new manuscript be written from a new angle that was not adequately covered in the past reviews and should also incorporate new studies that have accumulated since the last review(s). In addition, the new review might also highlight the approaches, limitations, and conclusions of the past studies. But the authors must not be excessively critical of the past reviews as this is regarded by many authors as a sign of poor professionalism [3, 79]. Daft [79] emphasized that it is more important for a reviewer to state how their research builds on previous work instead of outright claiming that previous works are incompetent and inadequate. However, if a series of related papers on one topic have a common error or research flaw that needs rectification, the reviewer must point this out with the aim of moving the field forward [3]. Like every other scientific paper, the main body of a review article also needs to be consistent in style, for example, in the choice of passive vs. active voice and present vs. past tense. It is also important to note that tables and figures can serve as a powerful tool for highlighting key points in the body of the review, and they are now considered core elements of reviews. For more guidance and insights into what should make up the contents of a good review article, readers are also advised to get familiarised with the Boote and Beile [80] literature review scoring rubric as well as the review article checklist of Short [81].

### 5.8. Tables and Figures

An ideal review article should be logically structured and efficiently utilise illustrations, in the form of tables and figures, to convey the key findings and relationships in the study. According to Tay [13], illustrations often take a secondary role in review papers when compared to primary research papers which are focused on illustrations. However, illustrations are very important in review articles as they can serve as succinct means of communicating major findings and insights. Franzblau and Chung [82] pointed out that illustrations serve three major purposes in a scientific article: they simplify complex data and relationships for better understanding, they minimise reading time by summarising and bringing to focus on the key findings (or trends), and last, they help to reduce the overall word count. Hence, inserting and constructing illustrations in a review article is as meticulous as it is important. However, important decisions should be made on whether the charts, figures, or tables to be potentially inserted in the manuscript are indeed needed and how best to design them [83]. Illustrations should enhance the text while providing necessary information; thus, the information described in illustrations should not contradict that in the main text and should also not be a repetition of texts [84]. Furthermore, illustrations must be autonomous, meaning they ought to be intelligible without having to read the text portion of the manuscript; thus, the reader does not have to flip back and forth between the illustration and the main text in order to understand it [85]. It should be noted that tables or figures that directly reiterate the main text or contain extraneous information will only make a mess of the manuscript and discourage readers [86].

Kotz and Cals [87] recommend that the layout of tables and figures should be carefully designed in a clear manner with suitable layouts, which will allow them to be referred to logically and chronologically in the text. In addition, illustrations should only contain simple text, as lengthy details would contradict their initial objective, which was to provide simple examples or an overview. Furthermore, the use of abbreviations in illustrations, especially tables, should be avoided if possible. If not, the abbreviations should be defined explicitly in the footnotes or legends of the illustration [88]. Similarly, numerical values in tables and graphs should also be correctly approximated [84]. It is recommended that the number of tables and figures in the manuscript should not exceed the target journal's specification. According to Saver [89], they ideally should not account for more than one-third of the manuscript. Finally, the author(s) must seek permission and give credits for using an already published illustration when necessary. However, none of these are needed if the graphic is originally created by the author, but if it is a reproduced or an adapted illustration, the author must obtain permission from the copyright owner and include the necessary credit. One of the very important tools for designing illustrations is Creative Commons, a platform that provides a wide range of creative works which are available to the public for use and modification.

### 5.9. Conclusion/Future Perspectives

It has been observed that many reviews end abruptly with a short conclusion; however, a lot more can be included in this section in addition to what has been said in the major sections of the paper. Basically, the conclusion section of a review article should provide a summary of key findings from the main body of the manuscript. In this section, the author needs to revisit the critical points of the paper as well as highlight the accuracy, validity, and relevance of the inferences drawn in the article review. A good conclusion should highlight the relationship between the major points and the author's hypothesis as well as the relationship between the hypothesis and the broader discussion to demonstrate the significance of the review article in a larger context. In addition to giving a concise summary of the important findings that describe current knowledge, the conclusion must also offer a rationale for conducting future research [12]. Knowledge gaps should be identified, and themes should be logically developed in order to construct conceptual frameworks as well as present a way forward for future research in the field of study [11].

Furthermore, the author may have to justify the propositions made earlier in the manuscript, demonstrate how the paper extends past research works, and also suggest ways that the expounded theories can be empirically examined [3]. Unlike experimental studies which can only draw either a positive conclusion or ambiguous failure to reject the null hypothesis, four possible conclusions can be drawn from review articles [1]. First, the theory/hypothesis propounded may be correct after being proven from current evidence; second, the hypothesis may not be explicitly proven but is most probably the best guess. The third conclusion is that the currently available evidence does not permit a confident conclusion or a best guess, while the last conclusion is that the theory or hypothesis is false [1]. It is important not to present new information in the conclusion section which has link whatsoever with the rest of the manuscript. According to Harris et al. [90], the conclusions should, in essence, answer the question: if a reader were to remember one thing about the review, what would it be?

### 5.10. References

As it has been noted in different parts of this paper, authors must give the required credit to any work or source(s) of information that was included in the review article. This must include the in-text citations in the main body of the paper and the corresponding entries in the reference list. Ideally, this full bibliographical list is the last part of the review article, and it should contain all the books, book chapters, journal articles, reports, and other media, which were utilised in the manuscript. It has been noted that most journals and publishers have their own specific referencing styles which are all derived from the more popular styles such as the American Psychological Association (APA), Chicago, Harvard, Modern Language Association (MLA), and Vancouver styles. However, all these styles may be categorised into either the parenthetical or numerical referencing style. Although a few journals do not have strict referencing rules, it is the responsibility of the author to reference according to the style and instructions of the journal. Omissions and errors must be avoided at all costs, and this can be easily achieved by going over the references many times for due diligence [11]. According to Cronin et al. [12], a separate file for references can be created, and any work used in the manuscript can be added to this list immediately after being cited in the text [12]. In recent times, the emergence of various referencing management software applications such as Endnote, RefWorks, Mendeley, and Zotero has even made referencing easier. The majority of these software applications require little technical expertise, and many of them are free to use, while others may require a subscription. It is imperative, however, that even after using these software packages, the author must manually curate the references during the final draft, in order to avoid any errors, since these programs are not impervious to errors, particularly formatting errors.

## 6. Concluding Remarks

Writing a review article is a skill that needs to be learned; it is a rigorous but rewarding endeavour as it can provide a useful platform to project the emerging researcher or postgraduate student into the gratifying world of publishing. Thus, the reviewer must develop the ability to think critically, spot patterns in a large volume of information, and must be invested in writing without tiring. The prospective author must also be inspired and dedicated to the successful completion of the article while also ensuring that the review article is not just a mere list or summary of previous research. It is also important that the review process must be focused on the literature and not on the authors; thus, overt criticism of existing research and personal aspersions must be avoided at all costs. All ideas, sentences, words, and illustrations should be constructed in a way to avoid plagiarism; basically, this can be achieved by paraphrasing, summarising, and giving the necessary acknowledgments. Currently, there are many tools to track and detect plagiarism in manuscripts, ensuring that they fall within a reasonable similarity index (which is typically 15% or lower for most journals). Although the more popular of these tools, such as Turnitin and iThenticate, are subscription-based, there are many freely available web-based options as well. An ideal review article is supposed to motivate the research topic and describe its key concepts while delineating the boundaries of research. In this regard, experience-based information on how to methodologically develop acceptable and impactful review articles has been detailed in this paper. Furthermore, for a beginner, this guide has detailed “the why” and “the how” of authoring a good scientific review article. However, the information in this paper may as a whole or in parts be also applicable to other fields of research and to other writing endeavours such as writing literature review in theses, dissertations, and primary research articles. Finally, the intending authors must put all the basic rules of scientific writing and writing in general into cognizance. A comprehensive study of the articles cited within this paper and other related articles focused on scientific writing will further enhance the ability of the motivated beginner to deliver a good review article.

## Data Availability

The data and materials that support the findings of this study are available from the corresponding author upon reasonable request.
